# The hypermucoviscosity of hypervirulent *K. pneumoniae* confers the ability to evade neutrophil-mediated phagocytosis

**DOI:** 10.1080/21505594.2021.1960101

**Published:** 2021-08-02

**Authors:** Qi Xu, Xuemei Yang, Edward Wai Chi Chan, Sheng Chen

**Affiliations:** a Department of Infectious Diseases and Public Health, Jockey Club College of Veterinary Medicine and Life Sciences, City University of Hong Kong, Kowloon, Hong Kong; b State Key Lab of Chemical Biology and Drug Discovery, Department of Applied Biology and Chemical Technology, The Hong Kong Polytechnic University, Kowloon, Hong Kong

**Keywords:** Hypervirulent *K. pneumoniae*, capsule, hypermucoviscosity, neutrophil cells, phagocytosis

## Abstract

Hypervirulent *Klebsiella pneumoniae* (HvKP), which causes highly fatal infections, is a new threat to human health. In an attempt to investigate the underlying mechanisms of resistance to neutrophil-mediated killing and hence expression of high-level virulence by HvKP, we tested the binding affinity of HvKP strains to various types of human cells. Our data showed that HvKP exhibited weaker binding to both lung epithelial cells, intestinal Caco-2 cells and macrophages when compared to the classic, non-hypervirulent strains (cKP). Consistently, transconjugants that have acquired a *rmpA* or *rmpA2*-bearing plasmid were found to exhibit decreased adhesion to various types of human cells, and hence higher survival rate upon exposure to neutrophil cells. We further found that over production of hypermucoviscosity (HMV), but not capsular polysaccharide (CPS), contributed to the reduced binding and phagocytosis. The effect of hypermucoviscosity on enhancing HvKP virulence was further shown in human serum survival assays and animal experiments. Findings in this study therefore confirmed that *rmpA/A2*-mediated hypermucoviscosity in HvKP plays a key role in the pathogenesis of this organism through conferring the ability to evade neutrophil binding and phagocytosis.

## Introduction

Hypervirulent *Klebsiella pneumoniae* (HvKP) has become a major pathogen that causes severe human infections such as pyogenic liver abscesses [1], pneumonia [2], and endophthalmitis [3]. HvKP strains typically possess a thick, hypermucoid capsule and therefore produce mucoid colonies that generate a positive result in string test [4,5]. HvKP exhibits features of enhanced virulence including production of a larger amount of capsule polysaccharide (CPS), ability to resist phagocytosis, and distant metastases [6]. The capsule is a key virulence factor that renders HvKP resistant to the complements and able to form a biofilm, which in turn confers resistance to antibiotics and starvation stress [7,8]. We recently showed that classical *K. pneumoniae* (cKP) strains could readily acquire the hypervirulence phenotype such as hypermucovisicosity and production of capsule saccharide through obtaining the virulence plasmid by conjugation [9,10]; such event results in a sharp increase in the incidence of HvKP infections.

The neutrophil-mediated response enables the human host to combat bacterial infection. Previous studies have shown that serotype K1 of HvKP is more resistant to neutrophil-mediated phagocytosis than non-K1 strains [11]. HvKP also exhibits a significantly higher level of resistance to neutrophil-mediated phagocytosis and intracellular killing when compared to cKP strains, which are more readily being retained in neutrophil extracellular traps (NETs) [12]. Two representative outbreak strains, *K. pneumoniae* 4 and 5 were found to exhibit significantly higher survival rate than classical ST11 strains in human neutrophil assay [13]. Understanding how capsulated HvKPs resist neutrophil-mediated killing is critical to the development of effective strategies to control infections caused by this important pathogen.

In this study, various clinical cKP and HvKP strains which express a variety of phenotypes regarding the degree of mucoviscosity and production of CPS were selected to investigate the underlying mechanisms of enhanced virulence of HvKP.

## Results

### Hypermucoviscous strains evade adhesion and phagocytosis

**Figure 1.**
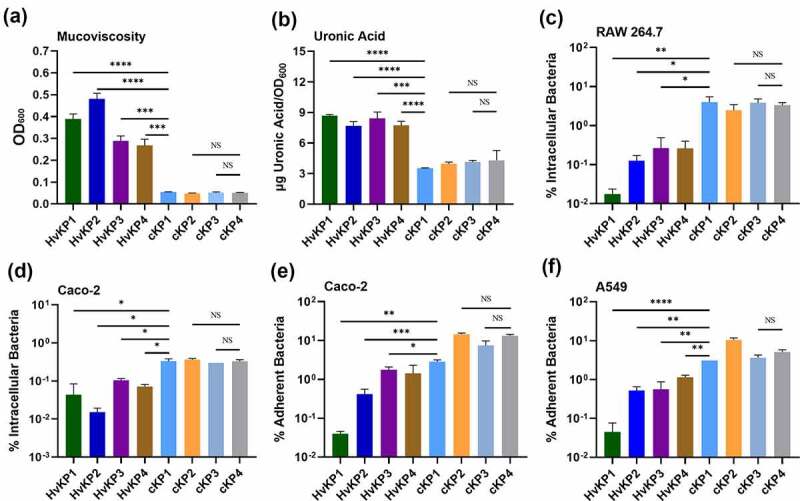
The hypermucoid phenotype of *K. pneumoniae* is associated with inhibition of cell adherence and internalization. Mucoviscosity (a) and uronic acid production (b) in hypervirulent *Klebsiella* strains HvKP1, HvKP2, HvKP3, HvKP4, and classical *Klebsiella* strains cKP1, cKP2, cKP3, cKP4. Invasion assay of selected clinical *K. pneumoniae* strains using Macrophage RAW 264.7 cells (c) and intestinal epithelial Caco-2 cells (d). Adhesion assay of selected strains using lung epithelial A549 cells (e) and Caco-2 cells (f). Data were analyzed by one-way ANOVA test. Each data point was repeated three times (*n* = 3). Data are presented as the mean ± s.e.m. * *P* < 0.05; ** *P* < 0.01; ***P < 0.001; **** *P* < 0.0001. Abbreviation: NS, not significant

Previous studies showed that the capsule of hypervirulent *K. pneumoniae* was essential for resistance to phagocytosis [14–17]. A K1 clinical isolate, SGH10, was found to exhibit reduced phagocytosis and adherence when compared to the Δ*wcaJ* and Δ*wzy* mutants, which have a defective cell envelope [18]. In this study, we tested several clinical HvKP strains and non-HvKP strains for their binding to different cell lines and phagocytosis by macrophage cells. Detailed genetic information of clinical HvKP strains tested in this study are listed in Table 1. Strains HvKP1, HvKP2, HvKP3, and HvKP4, exhibited a mucoid phenotype and produced a larger amount of uronic acid than the cKP strains cKP1, cKP2, cKP3, and cKP4 (Figure 1(a,b)). Consistently, HvKP1 and the other three HvKP strains were more resistant to uptake by RAW 264.7 when compared to the cKP strains (Figure 1(c)). It is known that the ability of bacteria to adhere to the intestines contributes to their persistence in the gut. However, HvKP strains with thicker capsule were found to exhibit weaker binding to both lung epithelial A549 cells and intestinal Caco-2 cells when compared to cKP strains (Figure 1(e,f)). Furthermore, HvKP strains were less likely to enter the Caco-2 cells when compared to cKP strains (Figure 1(d)). To visualize the binding and phagocytosis process, we expressed GFP protein in selected clinical *K. pneumoniae* strains, namely cKP4 and HvKP1, and tested the ability of these two strains to adhere to macrophage cells by fluorescence microscopy. The GFP-tagged cKP strain cKP4-GFP exhibited significantly increased potential to adhere to RAW 264.7 cells than the GFP-tagged HvKP1 strain HvKP1-GFP (Fig S4). Interestingly, some of the cKP4-GFP strains could be engulfed by macrophage cells during the incubation period. In contrast, few HvKP1-GFP strains adhered to the macrophages, and most maintained a distance from macrophage cells. Bacteria that adhered to the cells were numbered for normalization (Fig S5).

**Table 1.** Phenotypic and genotypic characteristics of *K. pneumoniae* strains used in this study

Strain Name	Strain ID used in this paper		STs	MIC [µg mL^−1^]												*rmpA*	*rmpA2*	String Test
				MEM		CTX		CAZ		AMK		CIP	TIG	PB	TE			
17ZR-101		HvKP1	ST86		32		> 128		> 128		4	4	0.25	4	> 128	**+**	truncated	**+**
HvKP1088[38]		HvKP2	ST23		64		< 0.06		128		2	64	0.5	2	> 128	**+**	truncated	**+**
GH22		HvKP3	ST23		< 0.06		< 0.06		2		2	< 0.06	0.25	4	> 128	**+**	truncated	**+**
EH78		HvKP4	ST23		< 0.06		< 0.06		0.25		1	< 0.06	0.25	4	> 128	**+**	truncated	**+**
17ZR-66		HvKP5	ST11		64		> 128		> 128		> 128	> 32	0.5	1	128	truncated	+	-
FJ8[39]		cKP1	ST11		32		64		> 128		128	64	1	4	2	-	-	-
HKU1		cKP2	ST716		0.06		> 128		8		128	8	4	2	4	-	-	-
KP04-1		cKP3	ST11		0.06		128		128		64	32	4	4	16	-	-	-
PM-28		cKP4	ST14		2		4		16		2	1	0.5	2	32	-	-	-
16HN-35		NP-HvKP	ST11		> 128		> 128		> 128		2	64	0.5	2	> 128	truncated	truncated	-
16HN-35/A		NP-HvKP /A	ST11		> 128		> 128		> 128		2	64	0.5	2	> 128	**+**	-	**+**
16HN-35/A2		NP-HvKP /A2	ST11		> 128		> 128		> 128		4	> 64	0.5	2	> 128	-	**+**	**+**
WZ1-2[9,10]		NP-HvKP-PC	ST11		64		> 128		32		2	64	0.5	2	0.5	-	-	-
WZ1-2-TC[9,10]		NP-HvKP-TC	ST11		>128		> 128		> 128		1	> 128	0.5	2	> 128	**+**	-	**+**

MEM, meropenem; CTX, cefotaxime; CAZ, ceftazidime; AMK, amikacin; CIP, ciprofloxacin; TIG, tigecycline; PB, polymyxinB(E); TE, tellurite.

“ – ” indicates that the experiment or analysis was not applicable to this strain.

### Phenotypic hypermucoviscosity (HMV), but not capsule over-production in HvKP contributed to reduced cell binding and phagocytosis

**Figure 2.**
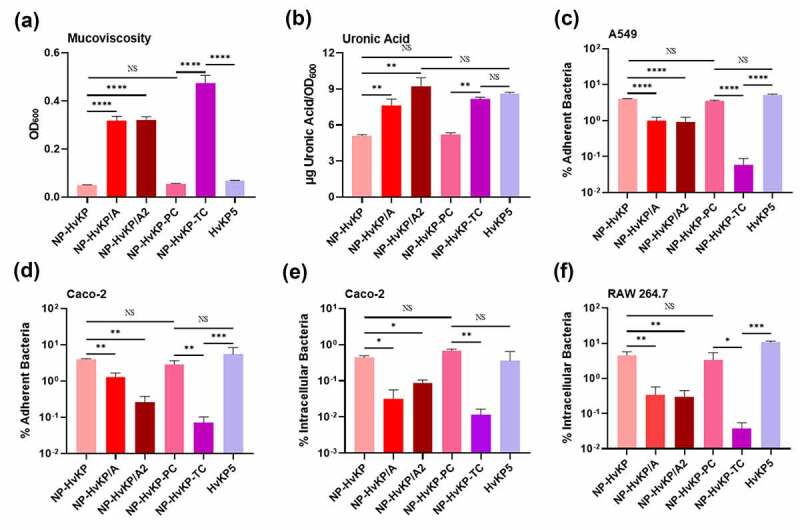
Cell adherence and internalization assay of *K. pneumoniae* strain NP-HvKP variants and HvKP5. Mucoviscosity (a), and uronic acid production (b) of *Klebsiella* strain NP-HvKP, NP-HvKP/A, NP-HvKP/A2, NP-HvKP-PC, NP-HvKP-TC and HvKP5. Assay of the ability of selected strains to adhere to lung epithelial A549 cells (c) and Caco-2 cells (d). Invasion assay of selected clinical *K. pneumoniae* strains using Macrophage RAW 264.7 cells (e) and intestinal epithelial Caco-2 cells (f). Data were analyzed by one-way ANOVA test. Each data point was repeated three times (*n* = 3). Data are presented as the mean ± s.e.m. * *P* < 0.05; ** *P* < 0.01; **** *P* < 0.0001. Abbreviation: NS, not significant

The hypermucoid nature of HvKP is known to be encoded by the regulator gene of the mucoid phenotype A (*rmpA*), which is typically located in the large virulence plasmid in HvKP, but may also be found in the chromosome [19–21]. Struve *et al* observed a close relationship between *rmpA* and expression of hypervirulence as all HvKP strains were *rmpA*-positive in their study [21]. A large virulence plasmid of CG43 contains two variants of *rmpA*, designated *rmpA* and *rmpA2*; both were shown to activate capsule production, resulting in the expression of a hypermucoviscous phenotype and increased virulence in mice [19,22]. To test the role of the virulence plasmid (*rmpA*/*rmpA2*) in capsule production, we selected one non-phenotypic HvKP strain, NP-HvKP, which carried a pLVPK-like virulence plasmid that contained truncated mutations in both *rmpA*/*rmpA2* genes (Table 1, Fig S2, Fig S3). Consistently, this strain did not exhibit HMV and high-level capsule production. Upon introducing a plasmid expressing RmpA (NP-HvKP/A) or RmpA2 (NP-HvKP/A2) into this strain, both transformants exhibited enhanced mucoviscosity and capsule production (Figure 2(a,b)). Taken together, these data indicate that the *rmpA* and *rmpA2* genes play a key role in the expression of the hypermucoid phenotype in this strain. Besides, we also created a virulence plasmid-complemented strain of NP-HvKP-PC as another positive-control strain, namely NP-HvKP-TC, in subsequent tests [10]. Mucoviscosity assay and uronic acid production assays showed that NP-HvKP-TC exhibited significantly increased mucoviscosity and production of capsule polysaccharide (uronic acid) when compared to the parental strain NP-HvKP-PC, suggesting that the virulence plasmid which contained the full-length *rmpA* gene contributed to HMV and CPS over-production.

Next, we examined cell adhesion and phagocytosis of the aforementioned *K. pneumoniae* strains. As expected, NP-HvKP/A and NP-HvKP/A2 exhibited decreased adhesion to A549 cells and Caco-2 cells (Figure 2(c,d)). Upon acquisition of the virulence plasmid, the transconjugant of NP-HvKP, namely NP-HvKP-TC, was also significantly less adherent to lung and intestinal epithelial cells (Figure 2(c,d)). In contrast, these three strains exhibited better survival and higher dissemination potential, and were more resistant to uptake by Caco-2 cells and macrophage RAW 264.7 cells when compared to the parental strain NP-HvKP (Figure 2(e,f)). We further examined their binding to RAW 264.7 by fluorescence microscopy. NP-HvKP/A and NP-HvKP/A2 expressing the GFP protein, namely NP-HvKP/A-GFP and NP-HvKP/A2-GFP, exhibited reduced binding to macrophage cells when compared to their GFP-expressing parental strain NP-HvKP-GFP (**Fig S4, Fig S5**). Taken together, these data suggested that either *rmpA* or *rmpA2* contributed to the HMV, thus further reducing the binding affinity of the strain to macrophage cells and enhancing resistance to phagocytosis.

Recent study has suggested that HMV and CPS over production might be two separable traits [17]. In order to investigate whether HMV or the over production of capsule contribute to the lower cell adhesion and invasion, we searched for clinical strains which exhibited only enhanced capsule production but not HMV. Upon extensive screening, we found a strain, designated as HvKP5, which exhibited hyper-capsule production capability (Figure 2(b)) but not HMV (Figure 2(a)). The HvKP5 strain exhibited binding affinity at a level similar to the NP-HvKP strain, with ~10% adherence to A549 (Figure 2(c)) and Caco-2 (Figure 2(d)) cell lines, which was much higher than that of HvKP strains. A similar trend was observed for internalization of this strain to Caco-2 (Figure 2(e)) and RAW cells (Figure 2(f)). Since HvKP5 strain still produced high level of capsule in a way resembling the HvKP strains, its higher level of binding to various cell lines was suggested to be due to the lower production of HMV. The data on HvKP5 strain further suggested that HMV is the major factor of HvKP that blocked both adherence and internalization.

### In vitro *and* in vivo *killing effect of different* K. pneumoniae *strains with and without the hyperviscosity phenotype*

**Figure 3.**
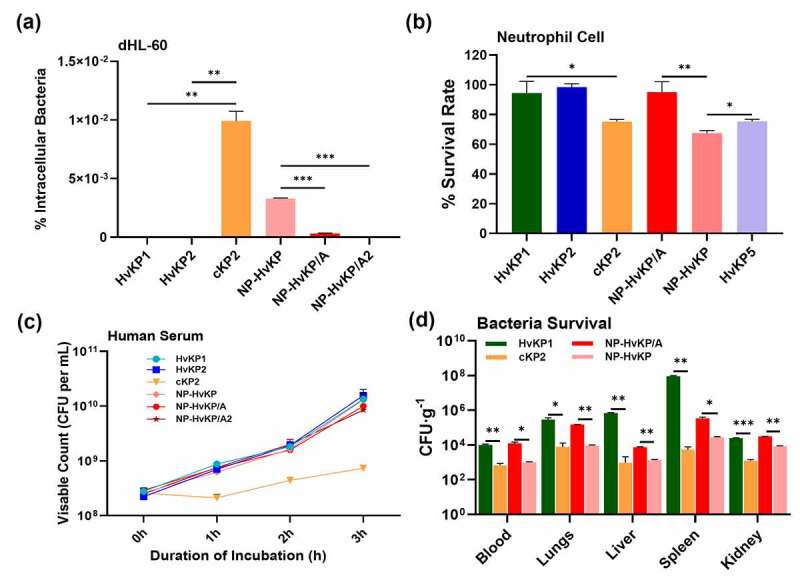
HvKP strains escape neutrophil-mediated phagocytosis and killing. (a) Neutrophil-like cell dHL-60 phagocytosis assay of HvKP1, HvKP2, cKP2, NP-HvKP, NP-HvKP/A, and NP-HvKP/A2. (b) Neutrophil killing assays of *Klebsiella pneumoniae* strains. (c) The resistance of *K. pneumoniae* strains to killing by pooled human serum. (d) Bacteria loads in blood, liver, lungs, kidney, and spleen were measured by plating at 24 hr post-infection. Bacterial load detectable in blood and various organs: HvKP1, cKP2, NP-HvKP/A and NP-HvKP. Data were analyzed by one-way ANOVA test. Each data point was repeated three times (n = 3). Data are presented as the mean ± s.e.m. * *P* < 0.05; ** *P* < 0.01; *** *P* < 0.001

Compared with classical *K. pneumoniae* infections, the hypervirulent *K. pneumoniae* strains caused much more severe pneumonia with a much higher mortality rate. Neutrophils are essential in the fight against bacterial infection. Hypervirulent *K. pneumoniae* strains have been reported to be more resistant to neutrophil-mediated killing than cKP strains [12,13]. We tested the sensitivity of phagocytosis of hypermucoviscous strain HvKP1, HvKP2, NP-HvKP/A, and NP-HvKP/A2, and non-hypermucoviscous strain cKP2 and NP-HvKP upon incubation with dHL-60 cells. Our results demonstrated that the non-hypermucoviscous strain cKP2 and NP-HvKP strains were more readily engulfed by dHL-60 than the hypermucoviscous strains (*p* < 0.05). No colonies of hypermucoviscous strain HvKP1, HvKP2 and NP-HvKP/A2 were detected inside the dHL-60 cells after 4 h incubation (Figure 3(a)). We further tested the survival fitness of the strains following incubation with neutrophil-like cells. Our data showed that the survival rate of hypermucoviscous strains HvKP1, HvKP2, NP-HvKP/A, NP/HvKP/A2 and HvKP5 which had been exposed to neutrophil cells and neutrophil-like dHL-60 cells was significantly higher than that of the cKP strain cKP2 and NP-HvKP (Figure 3(b), Fig S6). The data suggested that phenotypic resistance to phagocytosis exhibited by HvKP strains was due to their reduced binding to macrophage cells as a result of expression of the HMV phenotype.

Previous studies showed that human serum exhibited very limited bactericidal effect on most of *K. pneumoniae* strains [23]. The ability of clinical isolates used in this study to survive in human serum was investigated. Our data showed that human serum did not exhibit killing effect on all *K. pneumoniae* strains tested, but the growth rate of cKP strain was shown to be lower than HvKP strains in the presence of human serum (Figure 3(c)). The data suggested that immune cells rather than the complement system act as the defense barrier to *K. pneumoniae* at the beginning of the infection. The finding that these *K. pneumoniae* clinical isolates were susceptible to neutrophils but not human serum is consistent with our expectation that neutrophils, but not other components of the serum such as the complements, contribute to the observed bactericidal activity. The data further suggested that reduced phagocytosis due to HMV was the key mechanisms of virulence in HvKP strains.

The survival fitness of selected KP strains was also tested in the mouse model. ICR mice were infected by HvKP1 and cKP2 strains via tail vein injection. At 24 hr, the mice were sacrificed and the lungs, liver, spleen, and kidney were harvested, homogenized, and plated to enumerate the bacterial load of HvKP1 and cKP2 in each organ. Our result showed that strain HvKP1 could survive better in the bloodstream; in the organs the bacterial load was comparable with that of HvKP strains. The organ loads of the cKP strain cKP2 were significantly less than that of the HvKP1 strain, indicating that this strain is more susceptible to clearance by the immune system during systemic infection (Figure 3(d)). To confirm that *rmpA*-mediated mucoviscosity contributed to efficient dissemination of HvKP, the mice were also infected with NP-HvKP/A and NP-HvKP, respectively. As shown in Figure 3(d), NP-HvKP/A could spread efficiently in the bloodstream and the bacterial load in various organs was significantly higher than that of NP-HvKP strain. Taken together, these findings confirmed that *rmpA*-mediated mucoviscosity in HvKP plays a key role in the pathogenesis of this organism.

## Discussion

Several previous studies showed that HvKP strains could better survive upon being engulfed by neutrophils [12,13,24]. However, the molecular basis of how HvKP resists neutrophil-mediated killing is not understood and requires further investigation. The capsule of HvKP is a key virulence factor that aids dissemination and systemic infection in host [14,25–27]. The hypermucoid nature of HvKP is presumably contributed by the product of the *rmpA* and *rmpA2* genes, which are typically located in a large virulence plasmid harbored by HvKP [19–22]. Our *in vitro* adhesion assay suggested that the *K. pneumoniae* capsule renders the bacterial cell less adhesive to lung and intestinal epithelial cells. As such, we hypothesize that it is the thick and mucoviscous capsule that also renders HvKP strains resistant to phagocytosis by macrophage, neutrophil-mediated killing, and more efficient in dissemination in the body of the host. To test this hypothesis, we constructed two mutants, namely NP-HvKP/A and NP-HvKP/A2, which over-expressed the *rmpA* and *rmpA2* genes respectively. By comparing the phenotypes of these mutants in a different infection model, we sought to identify the changes that are attributed to RmpA or RmpA2-associated capsule production and expression of the mucoviscosity phenotype. Consistently, NP-HvKP/A and NP-HvKP/A2 exhibited reduced ability to adhere to the epithelial cells (Figure 2(c,d)) or undergo phagocytosis by macrophage (Figure 2(e)), as well as a lower chance of being killed by neutrophils (Figure 3(b)). Our data is consistent with previous reports that HvKP exhibited lower phagocytosis by macrophage when compared to cKP strains [18,28]. However, another study reported that deletion of *rmpA* (*∆rmpA*) in a HvKP strain, SGH10, exhibited no effect on uptake by RAW264.7 macrophages as well as adhesion to Caco-2 cells [18]. The data suggested that K. pneumoniae strain variability should be considered when measuring the binding and phagocytosis in cell lines.

Walker *et al* recently showed that, apart from the *rmpA* and *rmpA2* regulatory genes, HMV and over-production of CPS were mediated by two additional genes, namely *rmpC* and *rmpD* [17]. To confirm if HMV or over-production of CPS contributed to the reduced cell binding and phagocytosis, we selected one wild-type strain, HvKP5, which exhibited over-production of CPS but not HMV, for the binding and phagocytosis assays. Our data confirmed that HMV, but not over-production of CPS, contributed to reduced binding and phagocytosis. Our data is consistent with previous report in that knockout of the *rmpD* gene that encodes HMV, resulted in higher binding affinity to the host cells [16,17]. It is noted that our data only suggested that over production of CPS is not associated with reduced binding and phagocytosis, but it did not rule out the contribution of CPS at basal level to the cell binding and phagocytosis. It has been reported that the capsulated *K. pneumoniae* strains exhibited significantly higher level of binding to cell lines than their corresponding spontaneous noncapsulated variants suggesting the important role of CPS on cell binding [29,30]. The data from our study confirmed that HMV, while not the over production of CPS contributes to reduced cell binding and phagocytosis. The limitation of this study is the inability to generate isogenic gene knockout strains including *rmpA, rmpC* and *rmpD* knockout strains to confirm the contribution of HMV and CPS over production. The use of HvKP5 that exhibited overproduction of capsule but not HMV is acceptable, but not perfect since other genetic factors of HvKP5 that might affect the binding to neutrophil cells are not known. Therefore, studies using isogenic strains should be achieved to confirm the role of HMV and CPS over production in cell binding and phagocytosis in the future.

The complement system is the first line of defense against bacterial invaders that have breached the epithelial cell barrier of the host [23]. Resistance to complement is strongly correlated with survival, multiplication, and dissemination of a wide range of Gram-negative pathogens [31], and is a major virulence trait that enables *K. pneumoniae* to elicit invasive infections [32,33]. We also tested the survival fitness of HvKP and cKP strains in human serum and found that, although human serum did not exhibit killing effect on all *K. pneumoniae* strains tested, the growth rate of cKP strain was lower than HvKP strains in the presence of human serum (Figure 3(c)). The data suggested that immune cells rather than the complement system act as the defense barrier in *K. pneumoniae* killing at the beginning of the infection. Taken together, our work established the first model to demonstrate the underlying basis of the hypervirulence phenotype of HvKP strains (Fig S7). We showed that the *rmpA* and *rmpA2* genes are responsible for encoding the mucoviscosity phenotype, which is in turn essential for evasion of host immune defense and hence the persistence of HvKP in the bloodstream and systemic infection.

## Methods

### Bacterial strains and growth conditions

*Klebsiella* strains used in this study were recovered from clinical samples collected from different hospitals. These strains were available in our strain collection and listed in Table 1. A typical ST23 K1 HvKP strain 1088, which was demonstrated to be hypermucoviscous in our previous study, was used as a positive control and designated as HvKP2 in this study [9,10]. The genetic features of the HvKP virulence plasmid are shown in Supplementary Figure S1. A classic ST11 CRKP strain, FJ8, which was found to exhibit low mucoviscosity in our previous study, was used as a negative control and designated as cKP1^9^,^9^,[9, 10]. All strains were grown in Luria-Bertani (LB) medium at 37°C, with supplementation of 50 μg mL^−1^ kanamycin if needed.

### Mucoviscosity assay and string test

The mucoviscosity of the test *Klebsiella* strains was determined by performing the sedimentation assay as previously described [34]. Briefly, overnight culture grown in LB was diluted to an OD_600_ of 0.2 in media and grown at 37°C. At 6 h, the culture was normalized to an OD of 1.0 mL^−1^ and centrifuged for 5 min at 1000 × *g*. The supernatant was removed without disturbing the pellet for OD_600_ measurement. The string test was performed by stretching bacterial colonies grown on the sheep blood agar plate using an inoculation loop [5]. Results were presented as mean and standard deviation of data of three independent experiments.

### Extraction and quantification of capsule

Uronic acid was extracted and quantified as described previously [34], with slight modification. Briefly, the test strains were cultured for 6 h as described above. 500 µL culture were mixed with 100 µL capsule extraction buffer (100 mM citric acid, 1% Zwittergent 3–12), followed by incubation at 50°C for 20 min before centrifugation to pellet the cellular debris (5 min, 13,000 x *g*, room temperature). Capsule components were precipitated by incubating aliquots of supernatant (300 µL) with 1.2 mL absolute ethanol and centrifuged for 5 min at 13,000 x *g*. The pellet was air-dried and re‐suspended in 200 µL of sterile water, to which 1.2 mL of tetraborate solution (12.5 mM sodium tetraborate in sulfuric acid) was added and incubated for 5 min at 100°C, followed by immediate cooling on ice for at least 10 min. Uronic acid was detected by the addition of 20‐µL of hydroxyphenyl reagent (0.15% 3‐phenylphenol in 0.5% NaOH). After a 5‐min incubation at room temperature, the absorbance at 520 nm was measured. A standard curve constructed using glucuronic acid (Sigma-Aldrich) solutions of different concentrations was used to calculate the uronic acid concentration in the test samples. Results were presented as mean and standard deviation of data of three independent experiments.

### Phagocytosis of bacteria by macrophages

RAW 264.7 murine macrophages were grown in Dulbecco’s modified Eagle medium (DMEM) medium supplemented with 10% heat-inactivated fetal bovine serum (FBS). *K. pneumoniae* adherence and phagocytosis by macrophages were determined according to methods described previously, with slight modification [35]. Macrophages were seeded into 24-well tissue culture plates and infected with a multiplicity of infection of 50 (MOI 50; bacteria/cell) in a final volume of 1 mL DMEM. To synchronize the infection, plates were centrifuged at 200 × *g* for 5 min and then incubated at 37°C under a humidified 5% CO_2_ atmosphere. At 1.5 h, cells were rinsed three times with phosphate-buffered saline (PBS) and incubated for an additional 1.5 h with 1 mL DMEM and amikacin (300 µg mL^−1^) to eliminate extracellular bacteria. Cells were then rinsed again three times with PBS and lysed with 0.2% Triton X-100 (Sigma-Aldrich, St. Louis, MO, USA). Upon homogenization, 10-fold serial dilutions of the lysate were plated onto LB agar plates to determine the number of colony-forming units (CFUs) per unit volume.

### Adherence and invasion assays

Caco-2 human intestinal epithelial cells and A549 human lung epithelial cells were grown in DMEM supplemented with 10% heat-inactivated fetal bovine serum (FBS) and 1% nonessential amino acids (Gibco). The adherence assays and invasion assays of *K. pneumoniae* were performed as described previously [36,37]. In these assays, cells were seeded in 24-well plates overnight before *K. pneumoniae* infection. Cells in 24-well plates (~2.5 × 10^5^ cells per well) were prewashed with PBS. Mid-log-phase *K. pneumoniae* (OD_600_ = 0.4 to 0.6) in FBS-free DMEM medium were added to each well to achieve an MOI of 50. The number of CFUs inoculated per well was determined by serial dilution in PBS, plating on LB agar, and incubation for 12 h. For adherence assay, the infected plates were centrifuged for 5 min at 200 × *g* prior to the incubation to promote adherence of bacteria to cells; the plates were then incubated for 15 min in a humidified 5% CO_2_ atmosphere at 37°C. The wells were then washed three times with PBS, and adherence of bacteria to the wells was disrupted by the addition of 1 mL 0.2% Triton X-100. The adherence rate was the proportion of the inoculum that adhered to the wells of the plate.

To perform invasion assays, *K. pneumoniae* in DMEM medium containing 10% FBS were added to the wells, incubated for 2 h and washed three times with PBS, followed by a second incubation for 2 h with fresh medium containing 300 µg mL^−1^ amikacin; such antibiotic concentration was designed to kill extracellular bacteria according to the MIC listed in Table 1. Finally, the number of putative viable intracellular bacteria was determined by plating serial dilutions of the disrupted mixture onto LB agar and incubated for 12 h at 37°C. The invasion rate was the proportion of the inoculum that was internalized.

### *Construction of GFP-tagged* Klebsiella pneumoniae *strains*

The GFP-encoding gene was amplified using the GFP-F/GFP-R primer set and then cloned into the pCR2.1-TOPO vector to construct the pCR2.1-TOPO-GFP plasmid. Briefly, the GFP gene, was obtained by using the primer pair GFP-F (5ʹ-CGAGCTCGATGGTGAGCAAGG GCGAGG-3ʹ) and GFP-R (5ʹ-CGGGATCCTCAGTACTTGTACAGCTCGT-3ʹ) from the pBAVIK-T5-GFP plasmid (Addgene). The PCR products were digested by *BamH* I and *Sac* I restriction enzymes and ligated to the corresponding restriction sites of the pCR2.1-TOPO plasmid. The pCR2.1-TOPO-GFP plasmid was then transformed into *E. coli* DH5α cells.

For generation of GFP-tagged HvKP1 and cKP4, the pCR2.1-TOPO-GFP plasmid was transformed into these two strains, respectively. For generation of GFP-tagged NP-HvKP/A and NP-HvKP/A2, the *rmpA* and *rmpA2* gene from HvKP1088 and *K. pneumoniae* 4 [13] respectively were cloned, upon digestion by *Xba* I and *Spe* I, into pCR2.1-TOPO-GFP using the following primer pair: GFP-rmpA-F 5ʹ-CGAGATCTTTGACTGATGATTATTT-3ʹ and GFP-rmpA-R 5ʹ-CCTGATCACTAAATACTTGGCATGAGC-3ʹ for *rmpA*; GFP-rmpA2-F 5ʹ- CGAGATCTATGGAAAAATATATTTACTTTATG-3ʹ and GFP-rmpA2-R 5ʹ-CCTGATCACTAGGTATTTGATGTGCACC-3ʹ for *rmpA2*. All positive transformants were selected and the entire encoding sequence was determined to verify the absence of undesired mutations introduced by PCR. The plasmids pCR2.1-rmpA-GFP and pCR2.1-rmpA2-GFP were transformed into NP-HvKP to obtain the GFP-tagged NP-HvKP/A and NP-HvKP/A2, with kanamycin resistance gene as the selection marker.

### Fluorescence microscopy

Cells in 24-well plates (~2.0 × 10^5^ cells per well) were pre-washed with PBS three times. Mid-log-phase *K. pneumoniae* strains resuspended in DMEM medium were added to each well at an MOI of 50. After incubation for 15 mins, the wells were washed with PBS three times for the microscope. Adhered bacteria were also numbered for normalization.

### Neutrophil and dHL-60 phagocytosis and bacterial killing assay

The human leukemic cell line (HL-60) was maintained in the RPMI 1640 growth medium supplemented with 20% fetal bovine serum (FBS) at 37℃. For differentiation into neutrophil-like cells (dHL-60), the cells were cultured in the presence of 1.25% dimethyl sulfoxide for 3 days. The fresh neutrophil cell isolation was performed according to the protocol of the manufacturer’s instructions (Tbdscience, Tianjin, China). Neutrophils were infected with bacteria at an MOI of 50 and the infected plates were centrifuged for 4 min at 200 × *g* and then incubated at 37℃ with 5% CO_2_. Cells were infected for 2 h, then washed with PBS, and the culture medium was replaced by medium containing 300 µg mL^−1^ amikacin for a further 2 h incubation and lysed as described before. The number of putative viable intracellular bacteria was counted. For dHL-60 killing assay, the dHL-60 cells (5 × 10^5^) were challenged with *K. pneumoniae* at MOI 1:100 and incubated at 37℃ for 1 hin antibiotic-free RPMI medium. After incubation, the samples were collected and diluted to 10^1^ − 10^3^ bacteria mL^−1^. Relative survival rate was expressed as the number of CFUs recorded after neutrophil treatment when compared with the control.

For serum survival assay, bacteria were grown overnight in LB, sub-cultured 1:100 in fresh LB medium, and grown to late exponential phase (OD_600_ = 1). The culture was then subjected to centrifugation. The pellet was washed once in PBS and adjusted to OD_600_ = 0.15, diluted 1:10 in sterile phosphate-buffered saline; 50 µl of the diluted culture was added to 100 µl pre-warmed human serum (Sigma) and incubated at 37°C. Samples were collected at set time points, serially diluted, and plated for enumeration of viable bacteria.

### Animal experiments

All animal experiments were approved by the Animal Ethics Committee of the City University of Hong Kong and followed the guidelines of the Institutional Laboratory Animal Research Unit. For *in vivo* bacteria survival assays, mice were infected with 1 × 10^4^ CFU of HvKP and 1 × 10^4^ CFU of cKP, which were resuspended in 100 uL 1× PBS, via tail vein injection. At 24 h post-infection, the animals were sacrificed and serial dilutions of PBS-resuspended homogenized liver, lungs, kidney, and spleen samples were plated on MacCONKEY agar containing 20 μg mL^−1^ cefotaxime whose concentrations were fixed according to the minimal inhibitory concentration (MIC) (Table 1) recorded against the test strains. The bacterial loads in each sample was recorded.

### Statistical analysis

Data are presented as the mean ± the standard error of the mean. All graphical and numerical data were plotted using Graphpad Prism 6.0 (GraphPad Software, La Jolla California USA, www.graphpad.com). The statistical significance of differences between the samples and controls was determined using one-way ANOVA. *P* < 0.05 was used to determine whether the degree of difference was significant.

## Supplementary Material

Supplemental Material
