# Supplementary material for: The hypermucoviscosity of hypervirulent K. pneumoniae confers the ability to evade neutrophil-mediated phagocytosis
Source: Virulence. 2021 Aug 2;12(1):2050–9. doi: 10.1080/21505594.2021.1960101 (PMC8331041; doi:10.1080/21505594.2021.1960101)
Supplement: Supplemental Material [file KVIR_A_1960101_SM8319.zip › Supplementary_Material_R2.docx]

Supplementary materials

**The capsule of hypervirulent *K. pneumoniae* confers phenotypic hyperviscosity and ability to evade neutrophil-mediated phagocytosis**

Qi Xu^1,^ Xuemei Yang^1^, Edward Wai Chi Chan^2^, Sheng Chen^1*^

^1^ Department of Infectious Diseases and Public Health, Jockey Club College of Veterinary Medicine and Life Sciences, City University of Hong Kong, Kowloon, Hong Kong

^2^State Key Lab of Chemical Biology and Drug Discovery, Department of Applied Biology and Chemical Technology, The Hong Kong Polytechnic University, Hung Hom, Kowloon, Hong Kong

*For correspondence: Prof. Sheng Chen, [shechen@cityu.edu.hk](mailto:shechen@cityu.edu.hk)

**Keywords:** Hypervirulent *K. pneumoniae*, Capsule, Hyperviscosity, Nuetrophil, Phagocytosis


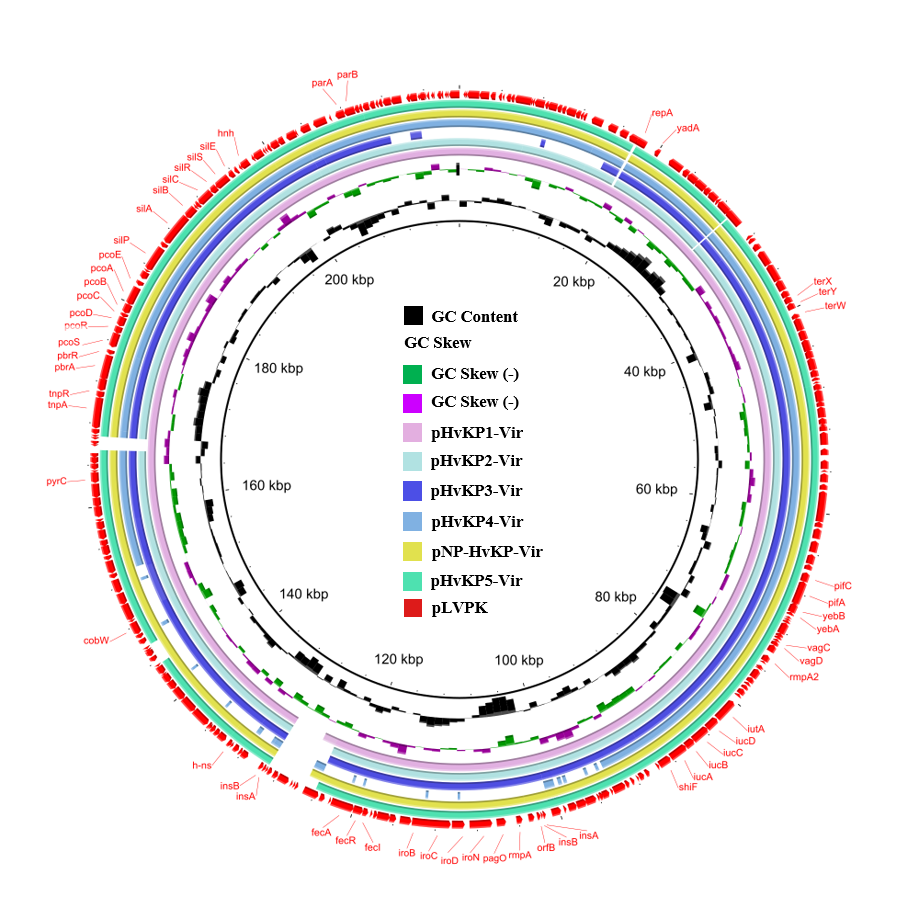


**Supplementary Figure S1.** Circular alignment of virulence plasmids studied in this work with pLVPK. All virulence plasmids tested carry the mucoid phenotype-encoding gene *rmpA* and *rmpA2*, although some of the genes exist in a truncated version as indicated in Table 1.


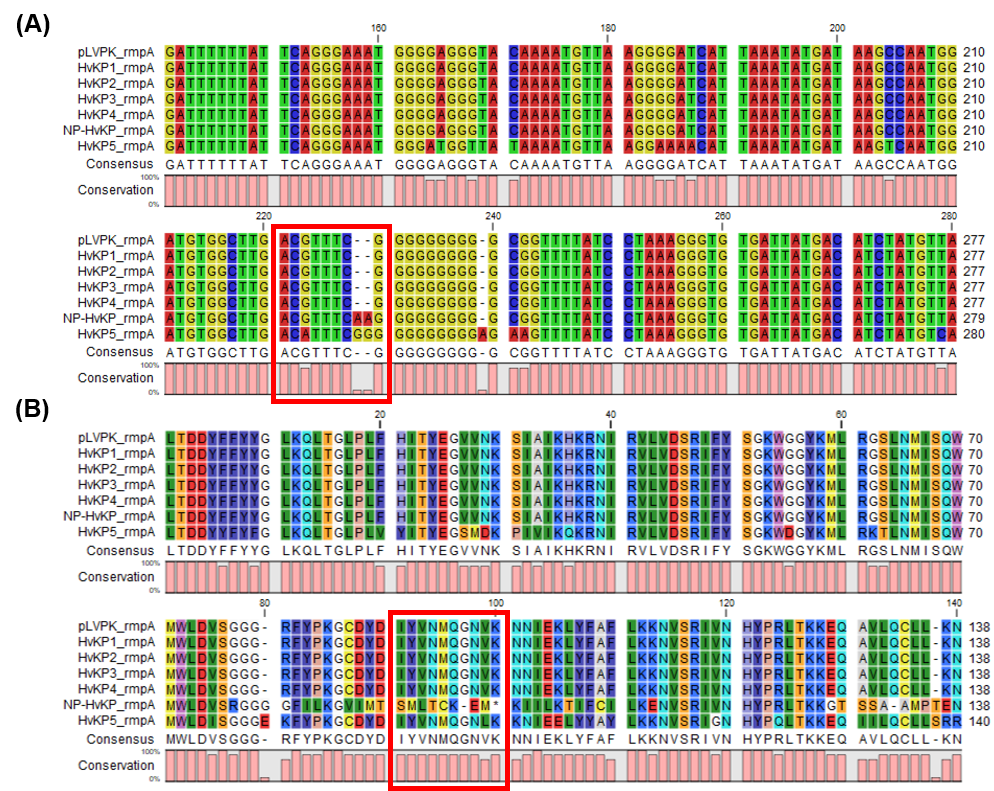


**Supplementary Figure S2.** *rmpA* gene and RmpA protein alignment of strain used in this study.

A. *rmpA* gene. B. RmpA protein. There are two As inserted at the site of 228 and 229 of NP-HvKP_*rmpA* (A) which resulted in a fragmentshift mutation on the site of 77 (B).


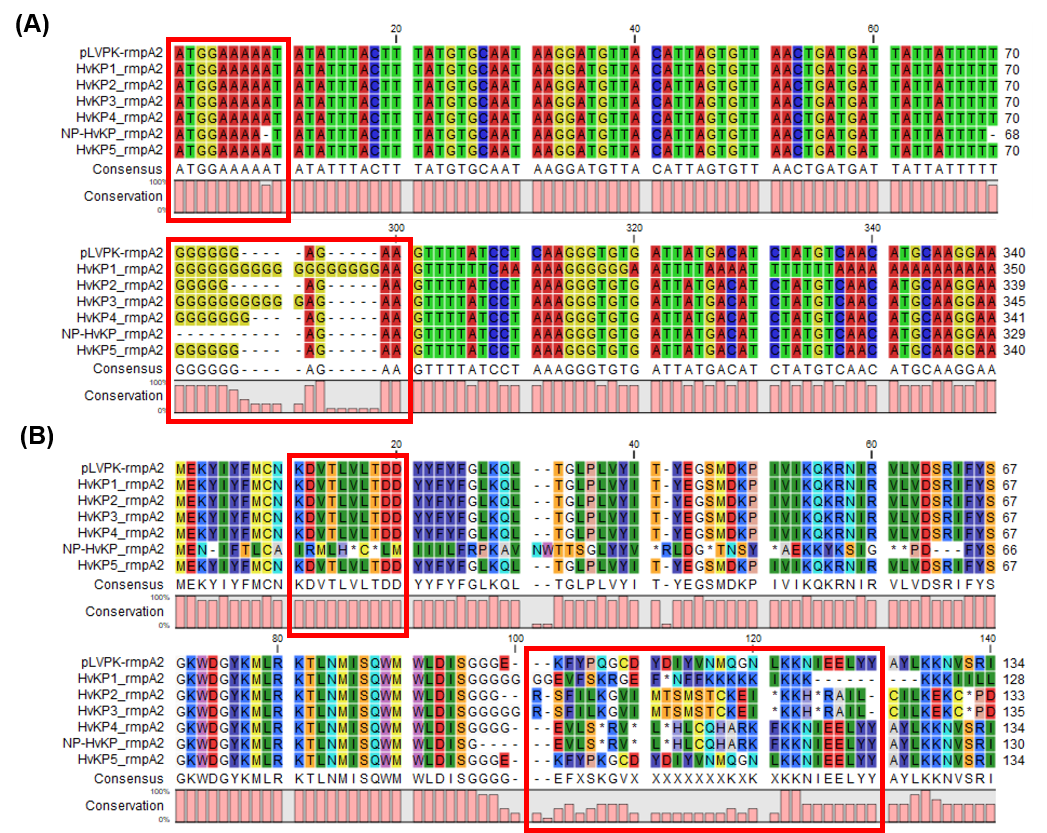


**Supplementary Figure S3.** *rmpA2* gene and RmpA2 protein alignment of strain used in this study. A. *rmpA* gene. B. RmpA protein. There is one A lost at the site of 9 of NP-HvKP_*rmpA* and several Gs inserted at the indicated site of HvKP1 – HvKP4 (A), which all resulted in a fragmentshift mutation at the different site (B).


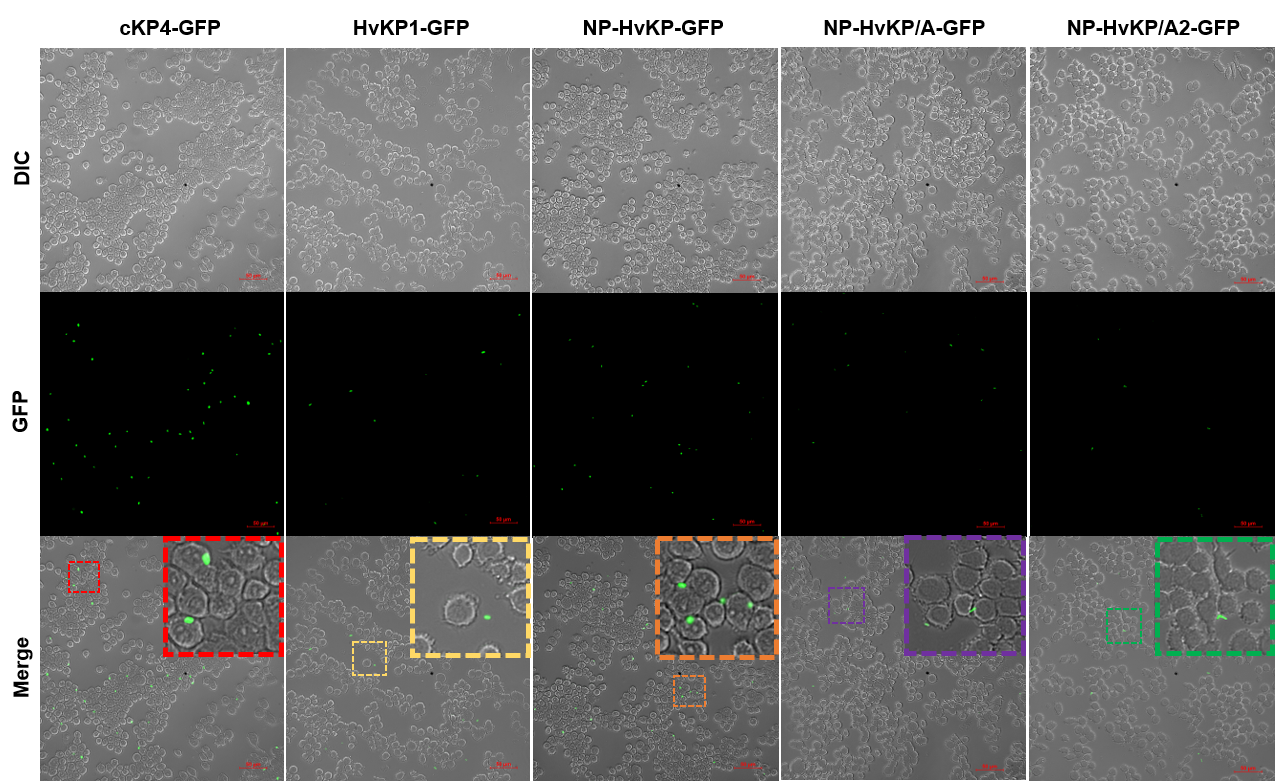


**Supplementary Figure S4. Fluorescence microscopy of *K. pneumoniae* binding to macrophage.** Fluorescence microscopy of GFP-tagged cKP4 (cKP4-GFP), HvKP1 (HvKP1-GFP), NP-HvKP (NP-HvKP-GFP), NP-HvKP/A (NP-HvKP/A-GFP), and NP-HvKP/A2 (NP-HvKP/A2-GFP) adhesion to macrophage RAW 264.7 cells.


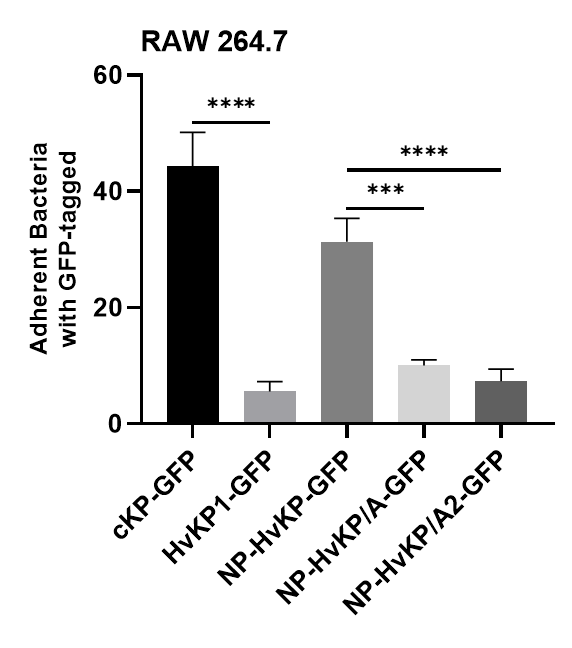


**Supplementary Figure S5. Number of adhered bacteria observable under a fluorescence microscope for normalization.** The cKP strain cKP4 and HvKP strain HvKP1 were used to investigate if hvpermucoviscous strains impede adhesion. Clinical strain NP-HvKP, whose virulence plasmid pNP-HvKP-Vir has both *rmpA* and *rmpA2* truncated, and strains NP-HvKP/A and NP-HvKP/A2 overexpressing RmpA and RmpA2 respectively, were used to investigate the role of the mucoid regulator in the expression of the hypermucoviscosity phenotype. Data were analyzed by one-way ANOVA test. Each data point was repeated three times (*n*=3). Data are presented as the mean±s.e.m. *** *P* < 0.001; **** *P* < 0.0001.

***
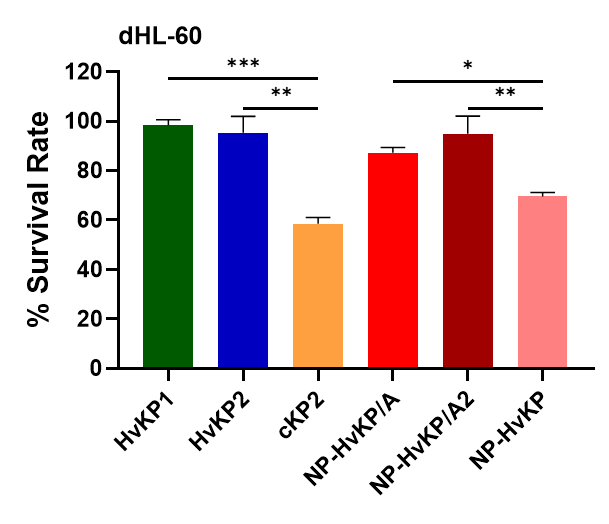
***

**Supplementary Figure S6.** dHL-60 killing assays of Klebsiella pneumoniae strains. Data were analyzed by one-way ANOVA test. Each data point was repeated three times (*n*=3). Data are presented as the mean±s.e.m. *** *P* < 0.001; **** *P* < 0.0001***.***


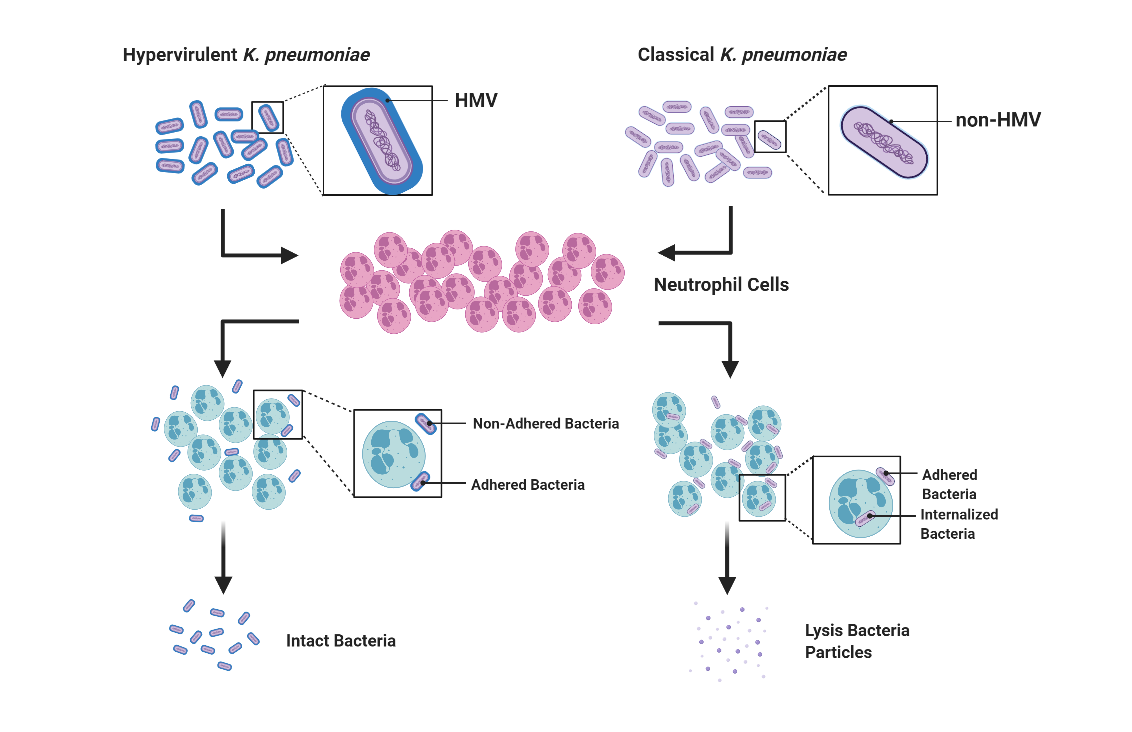


**Supplementary Figure S7. Schematic illustration of how hypervirulent *Klebsiella pneumoniae* escapes human neutrophil-mediated killing.** Compared with cKP strains which were engulfed or killed by neutrophil cells, the HMV of HvKP rendered it binding to and phagocyted by macrophage cells, resulting in better survival.
